# Development of PUFF–Gaussian dispersion model for the prediction of atmospheric distribution of particle concentration

**DOI:** 10.1038/s41598-021-86039-y

**Published:** 2021-03-19

**Authors:** Jooyong Lee, Sungsu Lee, HyunA Son, Waon-ho Yi

**Affiliations:** 1 Department of Disaster Prevention Engineering, Chungbuk National University, Cheongju, South Korea; 2 School of Civil Engineering, Chungbuk National University, Cheongju, South Korea; 3 Daewoo Shipbuilding and Marine Engineering, Geoje-Si, South Korea; 4 Department of Architectural Engineering, Kwangwoon University, Seoul, South Korea

**Keywords:** Environmental sciences, Natural hazards

## Abstract

Mt. Baekdu’s eruption precursors are continuously observed and have become a global social issue. Volcanic activities in neighboring Japan are also active. There are no direct risks of proximity-related disasters in South Korea from the volcanic eruptions at Japan or Mt. Baekdu; however, severe impacts are expected from the spread of volcanic ash. Numerical analysis models are generally used to predict and analyze the diffusion of volcanic ash, and each numerical analysis model has its own limitations caused by the computational algorithm it employs. In this study, we analyzed the PUFF–UAF model, an ash dispersion model based on the Lagrangian approach, and observed that the number of particles used in tracking substantially affected the results. Even with the presence of millions of particles, the concentration of ash predicted by the PUFF–UAF model does not accurately represent the dispersion. To overcome this deficit and utilize the computational efficiency of the Lagrangian model, we developed a PUFF–Gaussian model to consider the dispersive nature of ash by applying the Gaussian dispersion theory to the results of the PUFF–UAF model. The results of the proposed method were compared with the field measurements from actual volcanic eruptions, and the comparison showed that the proposed method can produce reasonably accurate predictions for ash dispersion.

## Introduction

Recently, warnings of potential volcanic eruptions have increased; these alerts have become a social issue, and importance of managing the disasters due to eruptions is also intensifying. Historically, Mt. Baekdu has acted as a volcanic eruption precursor suggesting the possibility of eruption with a strong eruption record corresponding to the volcanic eruption index of 7^1^. Moreover, Japanese volcanoes, which are close to Korea, have erupted more than 300 times within less than 1000 km and more than 40 times within 500 km of Seoul, over the past 50 years^2^. The volcanoes that have erupted within 500 km are especially similar to Mt. Baekdu, and future eruptions from these landforms may substantially impact Korea based on the volcanic eruption index and associated weather conditions. If these volcanoes erupt in the future, no direct damage (lava, lahar, etc.,) is expected, but the spread of volcanic ash may cause severe destruction.

Damage caused by volcanic ash spread can occur in sectors such as aviation, air, health, and agricultural and livestock products^3, 4^. For estimating the damage of volcanic ash dispersion following an eruption, volcanic ash diffusion predictions using numerical models, such as HYSPLIT^5^, FALL3D^6^, and PUFF–UAF, have been performed. A study of risk maps with the probabilistic analyses of impacts was performed^7, 8^. Globally, the Volcanic Ash Advisory Center (VAAC) performs volcanic eruption observations on major air routes around the world using numerical models. In a previous study, numerical and risk analyses of volcanic ash diffusion for domestic and Japanese volcanoes were performed using the PUFF–UAF model. However, inaccuracies measuring in volcanic ash concentrations derived from Lagrangian-based PUFF–UAF models have been noted^8^. On the other hand, particle diffusion analysis methods based on the Eulerian method, such as FALL 3D, are known to be relatively accurate in concentration predictions, but require considerably greater computational resources compared to the Lagrangian-based method^9^. Therefore, this study addresses the research aimed at developing a method that can improve the accuracy of concentration predictions by using the computational efficiency of the Lagrangian-based particle dispersion model. For this purpose, the PUFF–UAF model was first applied to the volcanic ash diffusion process to analyze concentration predictions by the Lagrangian-based particle diffusion model. Next, this study addresses the development of the PUFF–Gaussian model by applying the Gaussian dispersion theory that considers mass diffusion to compensate for the predicted concentration discontinuity. The PUFF–Gaussian model is then used to verify the developed method by predicting the volcanic ash diffusion concentration of volcanic eruptions.

## Lagrangian dispersion model by particle tracking

### PUFF–UAF model

The PUFF–UAF model is a volcanic ash tracking model based on the Lagrangian method, developed at Fairbanks University of Alaska, to simulate the spread of volcanic ash clouds^10^. In the early stages of development of the Lagrangian dispersion model, it was limited in the calculation area and the number of particles due to the large amount of time and system resources required for the calculation of a medium-sized area, at approximately 20–2000 km. However, its calculation time has been shortened, making it one of the fastest models in recent years.

The Lagrangian model sets the number of particles to an initial value to calculate the pollutant particle diffusion, and it is affected by the diffusion range and the concentration of the result.

The PUFF–UAF model uses wind data to track the diffusion motion of volcanic ash modeled into individual particles to perform volcanic ash diffusion predictions, and the volcanic ash particle position is calculated by Eq. (1) at each time step^10^.

1 $${R}_{i}\left(t+\Delta t\right)={R}_{i}\left(t\right)+W\left(t\right)\Delta t+Z\left(t\right)\Delta t+{S}_{i}(t)\Delta t$$

In Eq. (1), ${\mathrm{R}}_{i}$ is the position vector of the particle, $\mathrm{W}$ is the wind speed vector, $\mathrm{Z}$ is the turbulent diffusion term, and ${\mathrm{S}}_{\mathrm{i}}$ is the drop velocity vector due to gravity. In the above equation, the wind speed vector $\mathrm{W}$ can use various wind speed data such as NCEP and WRF, and the particle diffusion due to turbulence is considered by using the horizontal and vertical resolution applied to the calculation. In the PUFF–UAF model, the random walk model is introduced to consider the diffusion effect, and the decrease due to gravity acting on the particle is modeled by Stokes' law and applied as ${\mathrm{S}}_{\mathrm{i}}$ in Eq. (1).

**Figure 1:**
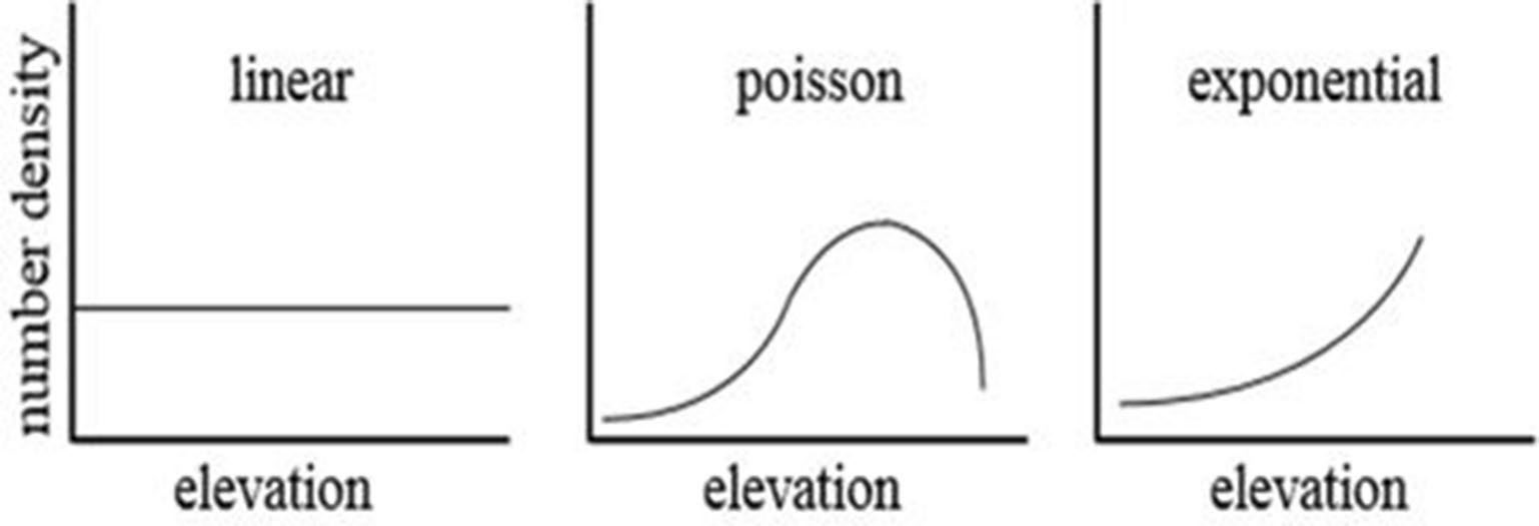
Density distribution model in PUFF–UAF^10^.

The PUFF–UAF model initializes particles at the volcanic crater location, and the initial position of each particle is placed from the height of the volcano crater to a random maximum ejection height. Consequently, the maximum ejection height is the input by the user referring to the volcanic explosivity index (VEI) or the measured value. It is also possible to place initial volcanic particles according to the altitude. As shown in Fig. 1, three different types of differentiation are provided: Poisson, Exponential, and Linear^10^. Generally, an exponential differentiation form is applied when VEI is 3 or less, and a Poisson eruption form is applied when a mushroom cloud form (mushroom type) or an umbrella cloud type (umbrella type) with VEI of 4 or more. If the intensity is unclear or cannot be confirmed, a numerical analysis is performed by applying the linear differentiation pattern.

Each ejected particle has independent ejection history information and is randomly released during the ejection time to obtain a result.

In addition to the density distribution along altitude, the particle size distribution is essential for the particle tracking. Since the data of the particle size distribution is hard to obtain for most of actual cases of eruption, the particle size distribution shown in Table 1 was used in this paper, which was obtained in the study for Etna volcano by Coltelli et al.^11^.

**Table 1:** Ash particle size distribution reported by Coltelli et al.^11^.

Diameter (mm)	Weight (%)
8	0.02
4	0.14
2	0.54
1	1.71
0.5	5.83
0.25	33.46
0.125	46
0.0625	11.15
0.02	1.14

### Concentration prediction

Lagrangian particle tracking methods, such as the PUFF–UAF model, initially represent the physical properties (i.e., mass and size) of the ejected particles that are transported through the wind and diffusion process described in the previous section. At each time step, the location of the particles is determined, which can be used to estimate the concentration of particles in the atmosphere.

**Figure 2:**
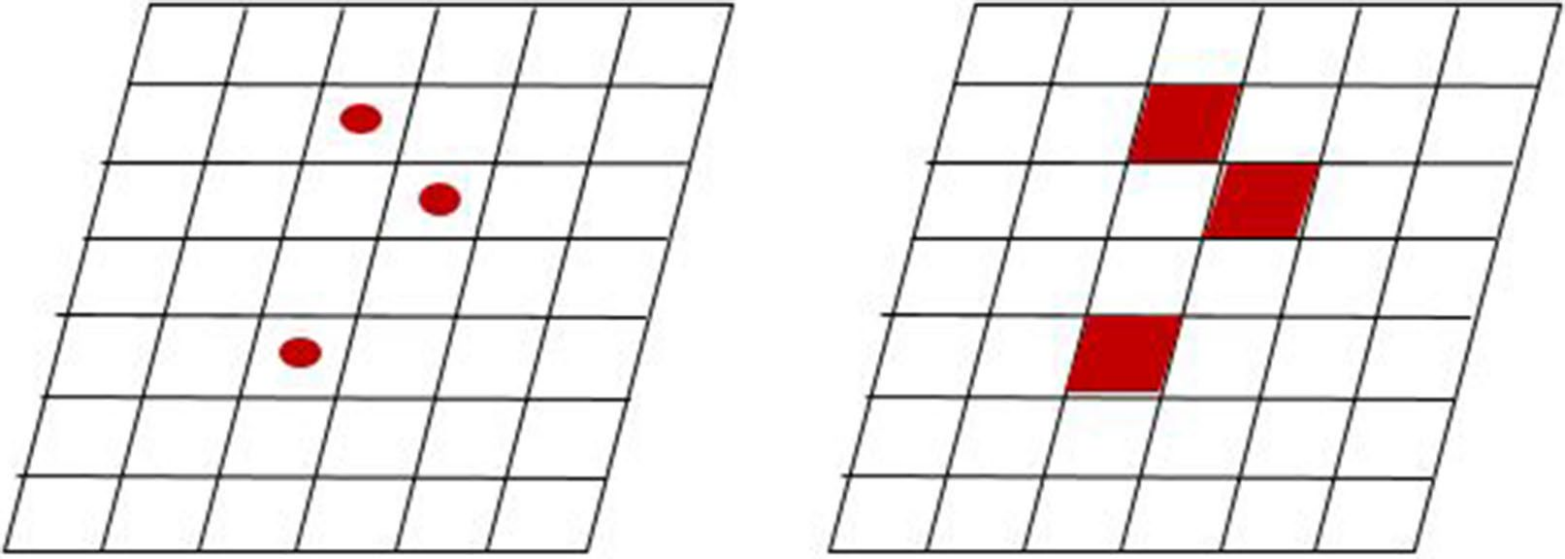
Estimation of particle concentration from the Lagrangian particle tracking method: (Left) Original locations of particles. (Right) Estimated concentration based on grid.

Figure 2 shows a grid around particles located in space. The concentration is estimated using the volume of the lattice in which the particles are located and the mass of the particles. In summary, when the volume of the space lattice is V and the mass of the particles located in the space is m_i_, the particle concentration of the lattice is calculated as shown in Eq. (2).

2 $$\mathrm{c}=\frac{\sum_{i}^{N}{m}_{i}}{V}$$

where N is the number of particles located in the space grid. Therefore, when the initial particle number is small, the concentration value may be over-valued as the mass represented by each particle increases. In addition, the concentration distribution as well as the concentration value can be greatly affected by the concentration estimation using a randomly set spatial grid.

### Effect of initial particle number on concentration prediction

As pointed out above, the concentration prediction can be largely dependent on the number of particles in the particle tracking method. In order to demonstrate the effect of the number of particles on the concentration prediction, volcanic ash dispersion analysis was performed using the PUFF–UAF model for the Shinmoedake Volcano^12^ in Japan, which erupted on January 26, 2011. Shinmoedake Volcano is 1700 m high and located at 31.934° N latitude and 130.862° E longitude. Three recorded eruptions have occurred at this volcano, with the total eruption mass amounting to 21,000,000 tons; the information for mass, time, and plume height for each eruption is shown in Table 2^12^.

**Table 2:** Shinmoedake volcanic eruption information.

	1st eruption	2nd eruption	3rd eruption
Date	2011.01.26 16:00	2011.01.26 22:00	2011.01.27. 04:00
Erupt mass (ton)	9,000,000	12,000,000	9,000,000
Duration time (h)	3	4	3
Plume height (m)	7000	3000	7000

The meteorological data used in the calculation is NCEP reanalysis data at 6-h intervals and has a horizontal grid resolution of 2.5° × 2.5° and a vertical resolution of 17 pressure levels (mb).

The grid resolution of the PUFF-UAF model is 0.1° × 0.1° horizontally, and the grid is calculated by generating 60 grids each 500 m vertically, and the input distribution by size of volcanic ash particles is set as shown in Table 1 because information on the actual particle size distribution erupted from Shinmoedake volcano is unknown.

To compare the effect of the initial particle number on the concentration distribution, a differentiation was simulated for two cases of initial particle numbers of 1,000,000 (Case 1) and 100,000 (Case 2), respectively. For the identical parameters of VEI^13^, i.e., the total eruption mass and ash particle size distribution, 10 times of difference in the number of initial particles was set to assess the effect of to the number of particles set at the beginning. In both cases, the distribution of particles in the periphery was assumed to be linear, and the result of the volcanic ash diffusion was saved every hour.

**Figure 3:**
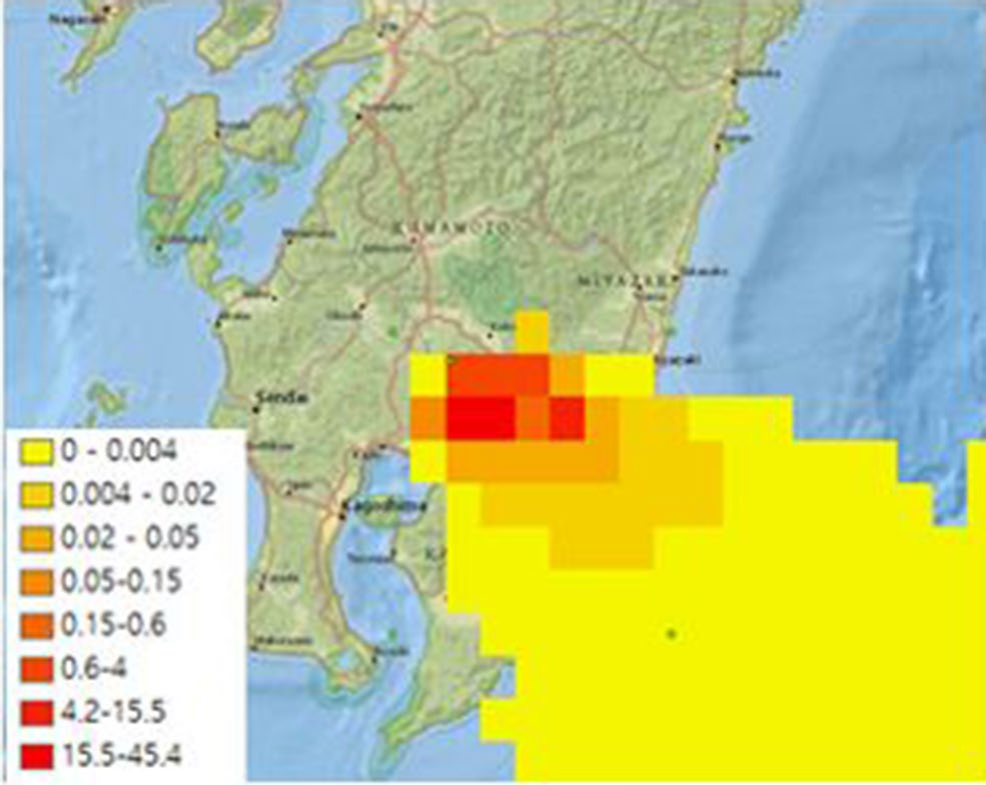
Ash concentration near ground and deposition by PUFF–UAF (Case1).

**Figure 4:**
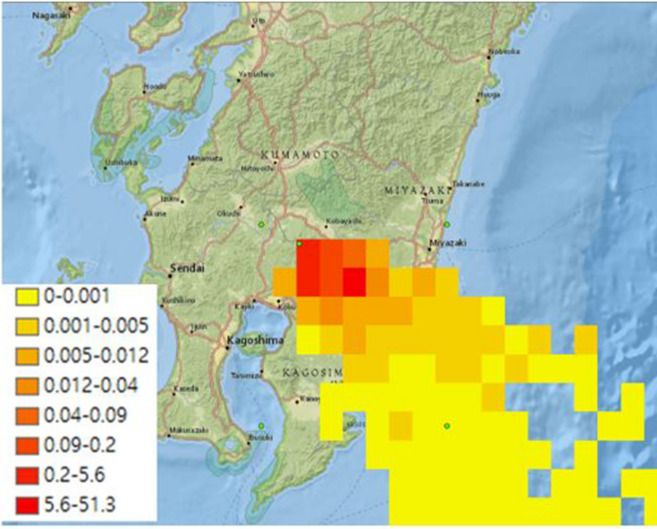
Ash concentration near ground and deposition by PUFF–UAF (Case2).

Figures 3 and 4 show the comparison of ash concentrations of Cases 1 and 2 in the atmosphere corresponding to the altitude of 500 m from the earth's surface; both cases show similar main diffusion directions incurred by the direction of the dominant wind.

However, the volcanic ash dispersion ranges in cross wind direction and concentration distributions of Case 1 and Case 2 differed according to the number of volcanic ash particles initially set. While Case 1 with larger number of particles results in ash spreading evenly over a wide range, Case 2 with smaller number of particles shows narrow range of dispersion and discontinuity in the concentration distribution as predicted in the description above.

Additionally, the comparison of the maximum concentrations shown in Figs. 3 and 4 shows that the maximum concentration level is high when the number of initial particles is small, which is another limitation of the Lagrangian-based method described in the previous section.

Figures 3 and 4 utilized a computation environment such as ArcGIS, which is available at https://desktop.arcgis.com/en/arcmap/10.3/manage-data/netcdf/ by first author of this paper.

As shown in the above results, the Lagrangian particle tracking model may produce a narrow diffusion range and relatively high concentration in localized region when the initial particle number is insufficient while it has advantages such as efficiency of short calculation time and prompt assessment of primary direction of particle dispersion.

## PUFF–Gaussian dispersion model development

### Basic ideas

As described in the previous section, concentration predictions through Lagrangian method are highly dependent on the number of particles used for tracking and the spatial grid set. Specifically, Figs. 2 and 4 show that the discontinuity of the concentration distribution was inevitably caused by the spatial grid set and the position of the particles.

Therefore, in this study, the following two assumptions were made to correct the possible results in the Lagrangian-based models:

1. Concentration diffusion or mass diffusion can also be applied to the discretized concentration distribution estimated by the Lagrangian method.
2. Mass diffusion follows the Gaussian dispersion model.

As a result, the authors developed a PUFF–Gaussian model that complements the results of the PUFF–UAF model.

### Gaussian dispersion model

**Figure 5:**
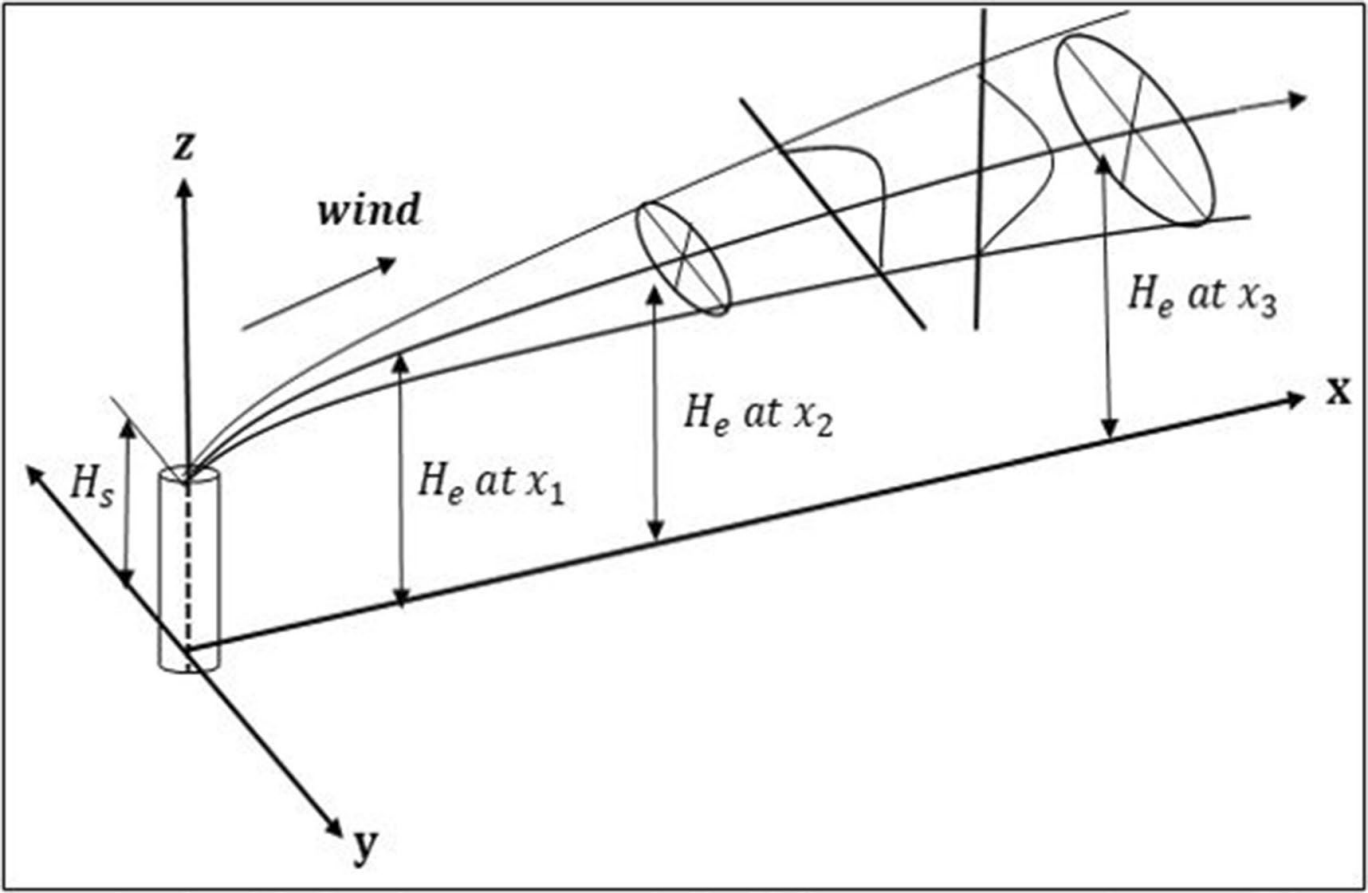
Gaussian dispersion model^13^.

The Gaussian dispersion model simulates the three-dimensional distribution of contaminant concentrations generated at a source, e.g., pollutants, at a specific location using fixed weather data and emission conditions employing Eq. (3)^14^.

3 $$\mathrm{C}\left(\mathrm{x},\mathrm{y},\mathrm{z}\right)= \frac{Q}{2\pi {\sigma }_{y}{\sigma }_{z}u}\mathrm{exp}\left(\frac{-{y}^{2}}{2{\sigma }_{y}^{2}}\right)\times [\mathrm{exp}\left(\frac{-{\left(z-h\right)}^{2}}{2{\sigma }_{z}^{2}}\right)+\mathrm{exp}\left(\frac{-{\left(z+h\right)}^{2}}{2{\sigma }_{z}^{2}}\right)]$$

where C is the concentration of a pollutant at a given position, Q is the emission term, x is the along-wind direction, y is the horizontal direction, z is the vertical direction, U is the wind speed, h is the height of the source, and ${\upsigma }_{\mathrm{y}}\mathrm{ and }{\upsigma }_{\mathrm{z}}$ represent the source variations in the horizontal and vertical directions, respectively. Equation (3) shows the mixing process of generating a Gaussian distribution in both the horizontal and vertical directions from the pollutant to the wind center line. This dynamic is expressed in Fig. 5^14^.

**Figure 6:**
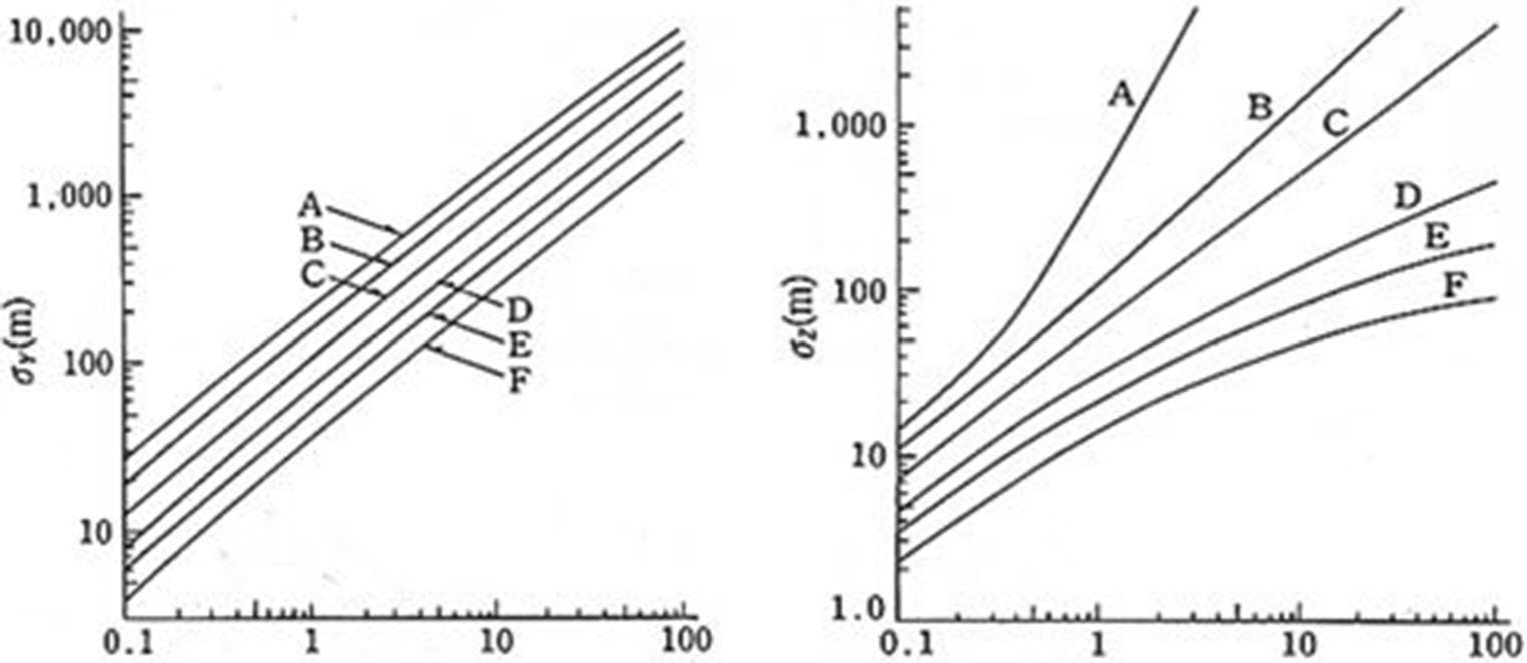
$${\upsigma }_{\mathrm{y}}\,\text{and}\,{\sigma }_{z}$$ values^13^.

The ${\upsigma }_{\mathrm{y}}$ and ${\upsigma }_{\mathrm{z}}$ used in Eq. (3) are variables that affect the degree of dispersion of pollutants emitted as a function of atmospheric stability and wind direction distance to the expected area, respectively. The sg and st used in Eq. (3) are variables that affect the degree of dispersion of pollutants emitted as a function of atmospheric stability and wind direction distance, respectively to the expected area of impact. The two factors that largely affect the degree of dispersion of emitted pollutants are the height of the source point and the degree of turbulence in the atmosphere, and high turbulence produces wide dispersion. Atmospheric vertical motion mixes different groupings of contaminated air, and the degree of vertical movement is determined by the atmospheric stability. The Gaussian dispersion model used in this study utilized Pasquill's atmospheric stability class to determine ${\upsigma }_{\mathrm{y}}$ and ${\upsigma }_{\mathrm{z}}$^14^. The relationship between the atmospheric stability class of Pasquill and ${\upsigma }_{\mathrm{y}}$ and ${\upsigma }_{\mathrm{z}}$ is shown in Fig. 6; the graph is approximated as in Eq. (4).

4 $${\upsigma }_{\mathrm{y}}=a{x}^{b} , {\sigma }_{Z}=c{x}^{d}+f$$

### PUFF–Gaussian model

**Figure 7:**
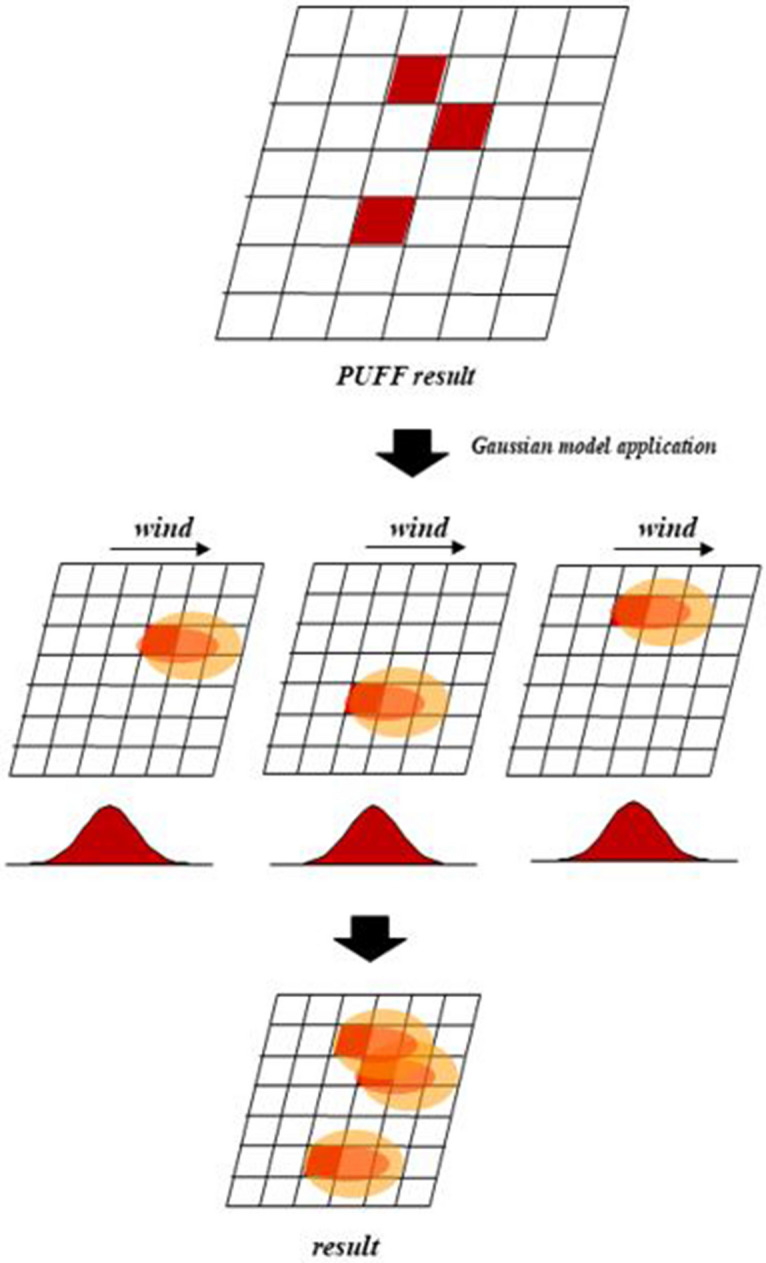
PUFF–Gaussian model concept.

Figure 7 illustrates the concept of the PUFF–Gaussian model developed in this study, and shows the process of correcting the results by diffusing the Gaussian dispersion model using the particle concentration in the spatial grid that is the result of the PUFF–UAF as a point source. Specifically, in Eq. (3) for the Gaussian dispersion, Q was calculated using the particle concentration diffused in each lattice of the result of the PUFF–UAF model, and u (wind velocity) was extracted from the weather data.

### Validation and verification of PUFF–Gaussian model

To validate and verify the PUFF-Gaussian model, the two actual eruptions of the Shinmoedake volcano and the Ontake volcano were tested, both cases of which the observation data of ash deposition on the ground are available.

First case was the eruption of the Shinmoedake volcano as specified in Table 2, which was employed above to demonstrate the effect of the particle numbers on the concentration prediction.

**Figure 8:**
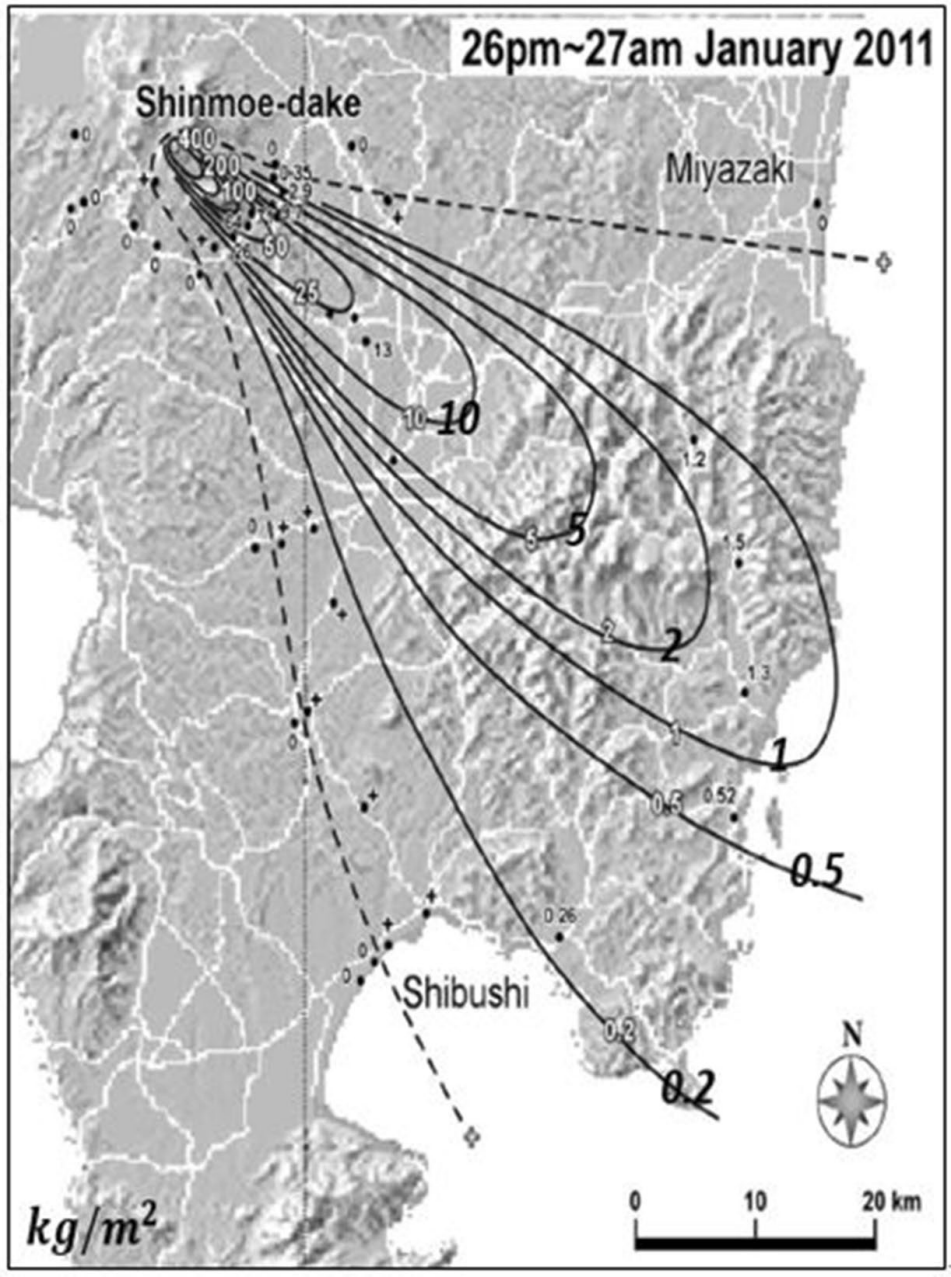
Observed ground deposition from the Shinmoedake volcano eruption from Jan. 26 to 27, 2011 (Maeno et al.^12^).

The Shinmoedake volcano, which began erupting on January 26, expelled ash widely in the southeast direction. Figure 8 shows the observation data of ash deposition on the ground^12^.

**Figure 9:**
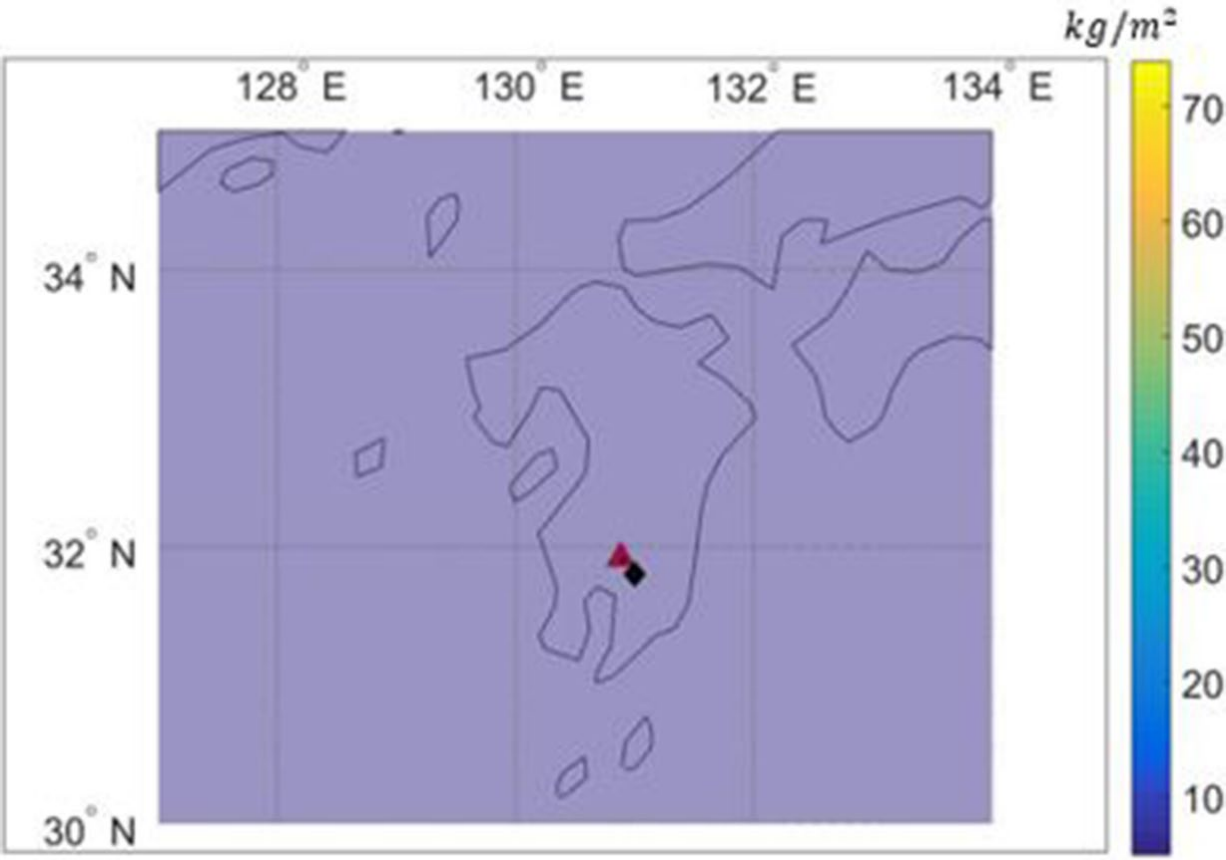
Prediction of ash deposition from the Shinmoedake volcano eruption from Jan. 26 to 27, 2011 by the PUFF–UAF model.

**Figure 10:**
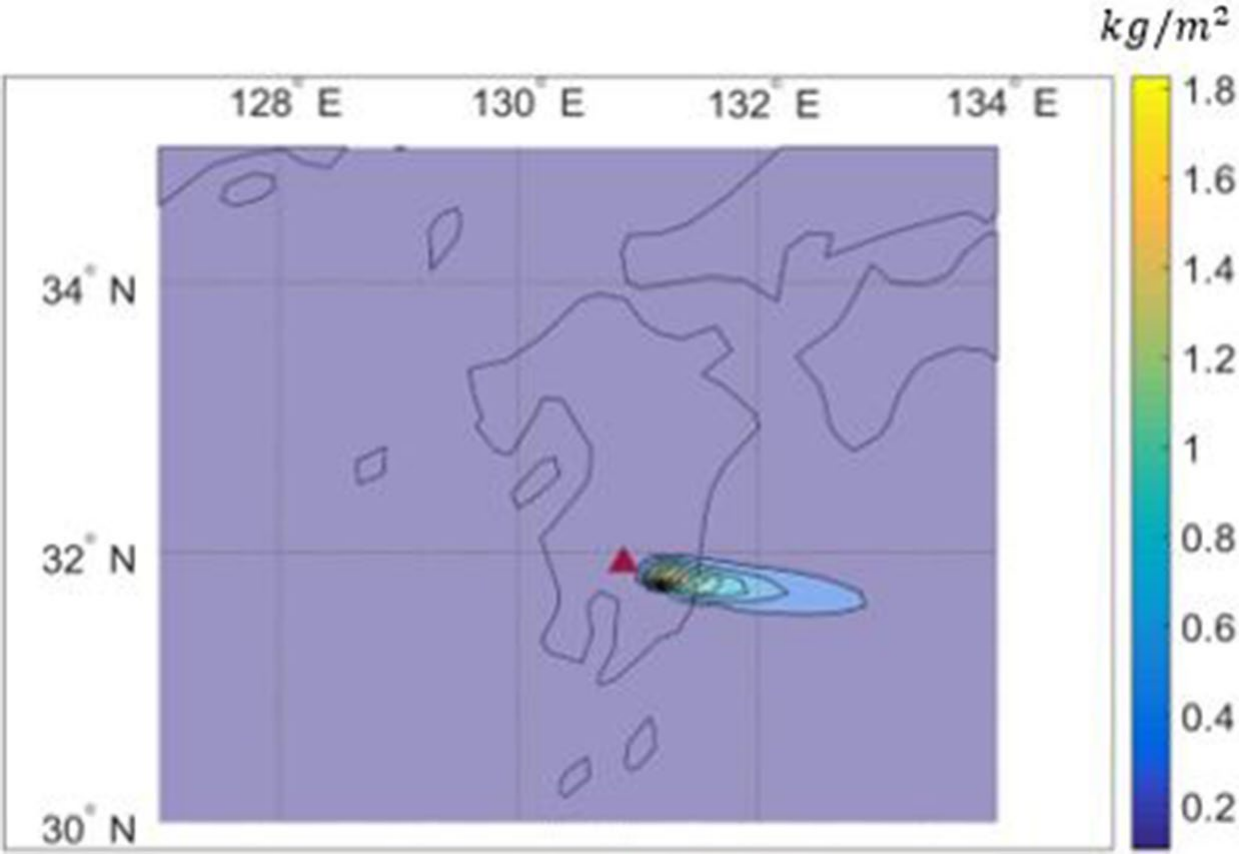
Prediction of ash deposition from the Shinmoedake volcano eruption from Jan. 26 to 27, 2011 by the PUFF–Gaussian model.

Figures 9 and 10 compares the distributions of ash deposition on the ground computed by PUFF–UAF and PUFF-Gaussian model. They clearly depict the validity of the present method by showing qualitative range of ash deposition on the ground.

**Figure 11:**
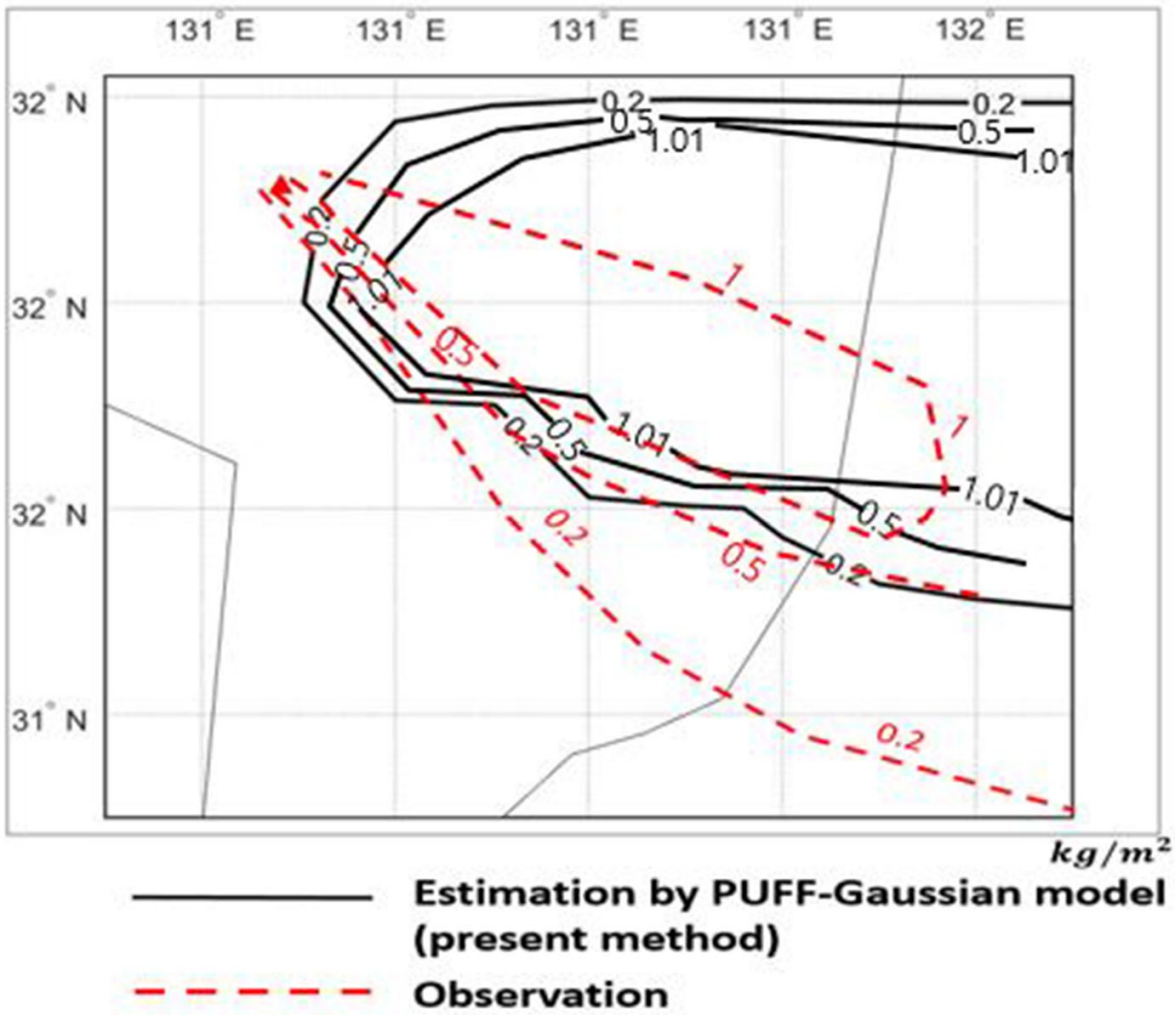
Comparison of ash deposition from the Shinmoedake volcano eruption from Jan. 26 to 27, 2011 between observations and the PUFF–Gaussian model for 0.2 $\mathrm{kg}/{\mathrm{m}}^{2}$, 0.5 $\mathrm{kg}/{\mathrm{m}}^{2},$ 1 $\mathrm{kg}/{\mathrm{m}}^{2}$.

The verification is made by comparing the observed ash depositions with and the computed results predicted by PUFF–Gaussian model in Fig. 11. The ranges of deposition as mass per unit area for comparison are 0.2 $\mathrm{kg}/{\mathrm{m}}^{2}$, 0.5 $\mathrm{kg}/{\mathrm{m}}^{2}$ and 1 $\mathrm{kg}/{\mathrm{m}}^{2}$ as shown in Fig. 11.

The results show that the present method predicted more concentrated region for higher deposition of 1 $\mathrm{kg}/{\mathrm{m}}^{2}$ compared to the observation while larger range in space is predicted for lower deposition of 0.2 $\mathrm{kg}/{\mathrm{m}}^{2}$. But overall distribution of the predicted deposition reasonably matches the observation.

**Figure 12:**
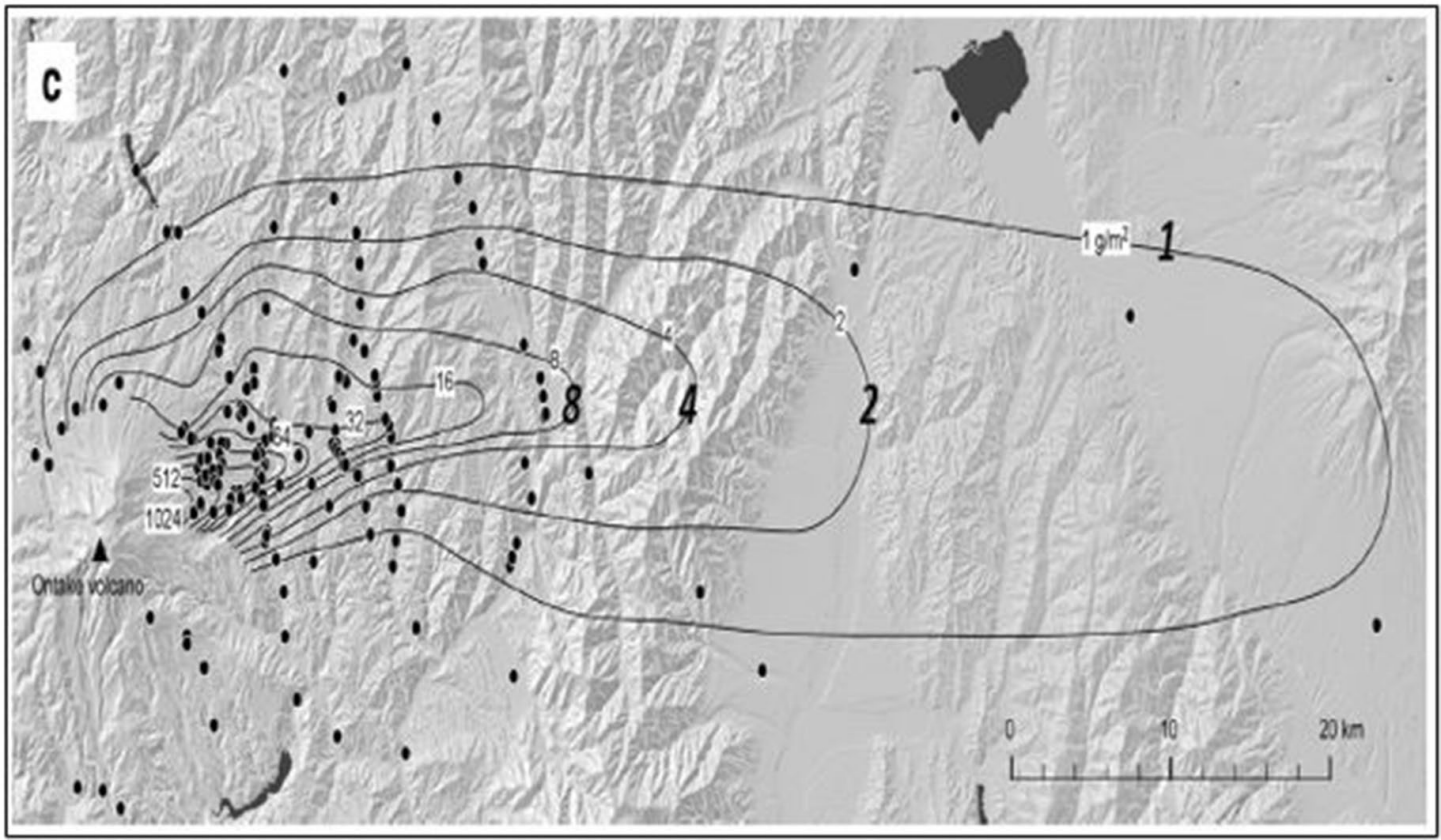
Deposition observations of Ontake volcanic eruption (Maeno et al.^15^).

The second case for the validation and the verification of the present method is the actual eruption of the Ontake volcano that began suddenly erupting on September 27, 2014. The observed plume height was about 5 km and the estimated total ejected volume was about 1.1 million ton. The observed distribution of ash deposition on the ground is shown in Fig. 12^15^.

**Figure 13:**
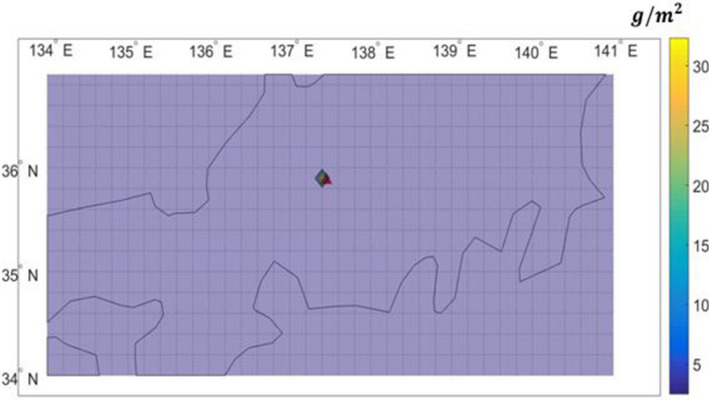
Deposition Results of PUFF–UAF Model (Ontake Volcano, February September 27, 2014).

**Figure 14:**
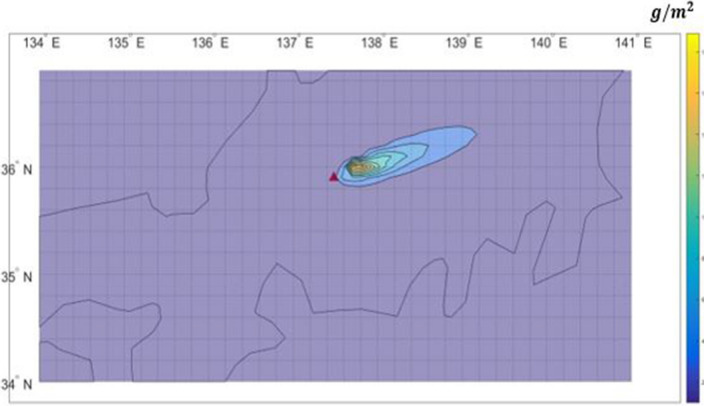
Deposition Results of PUFF–Gaussian Model (Ontake Volcano, February September 27, 2014).

Similarly, to the validation for the case of the Shinmoedake volcano, Figs. 13 and 14 also the qualitative validation showing smooth and continuous distribution of the deposition predicted by PUFF-Gaussian model.

**Figure 15:**
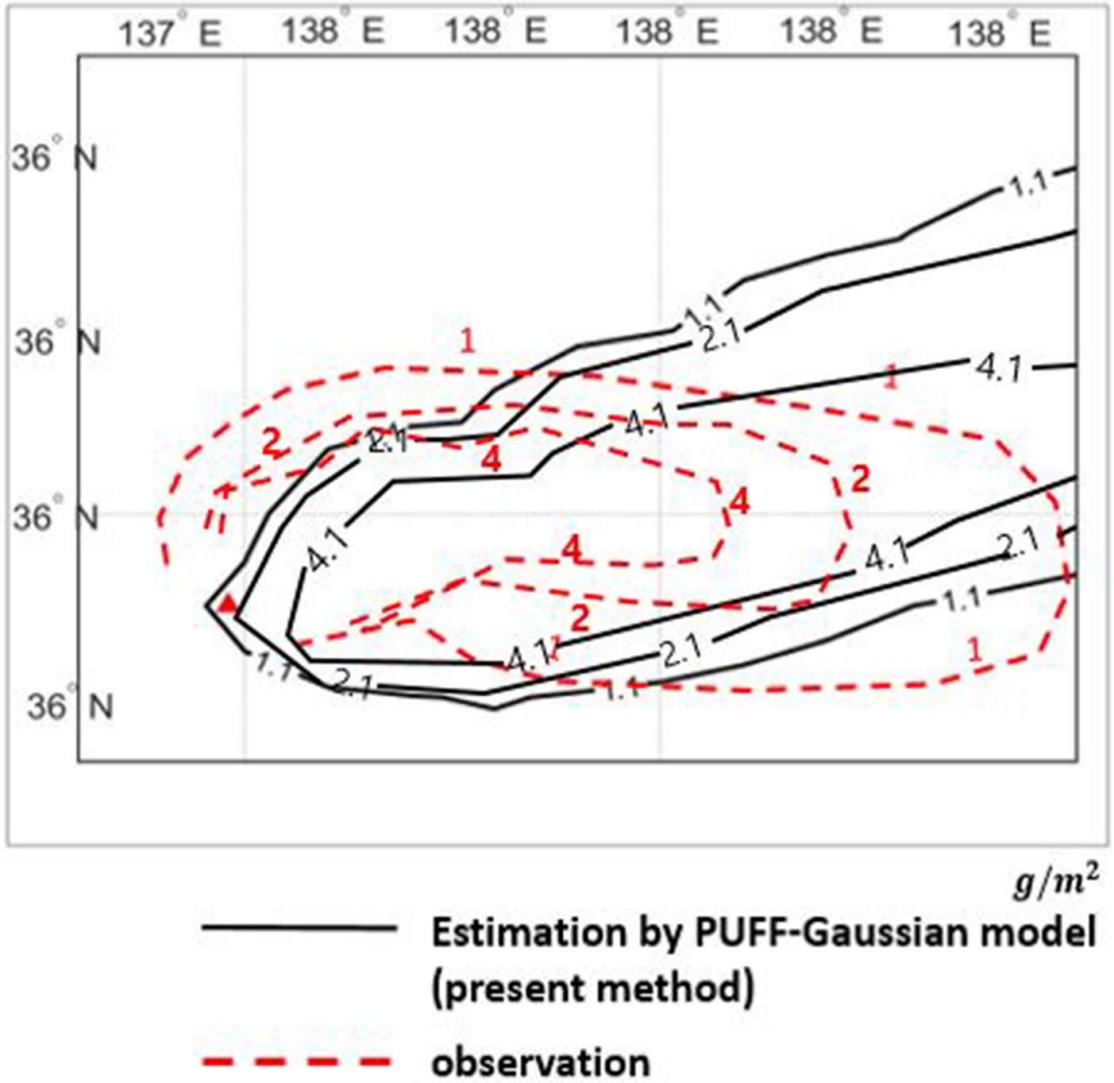
Comparison of ash deposition from the Ontake volcano eruption from September 27, 2014 between observations and the PUFF–Gaussian model estimation for 1 $\mathrm{g}/{\mathrm{m}}^{2}$, 2 $\mathrm{g}/{\mathrm{m}}^{2}$, 4 $\mathrm{g}/{\mathrm{m}}^{2}$

The verification is also made by comparing the observed ash depositions with and the computed results predicted by PUFF–Gaussian model in Fig. 15. The ranges of deposition as mass per unit area for comparison are 1 $\mathrm{g}/{\mathrm{m}}^{2}$, 2 $\mathrm{g}/{\mathrm{m}}^{2}$ and 4 $\mathrm{g}/{\mathrm{m}}^{2}$ as shown in Fig. 15.

The results show that the present method predicted more concentrated region for higher deposition of 2 $\mathrm{g}/{\mathrm{m}}^{2}$ and 4 $\mathrm{g}/{\mathrm{m}}^{2}$ compared to the observation. For 1 $\mathrm{g}/{\mathrm{m}}^{2}$ deposition range, the predicted deposition range of the PUFF–Gaussian model was similar to the observation and the diffusion range was slightly wider in the northeast direction. But overall distribution of the predicted deposition reasonably matches the observation.

### Further application of PUFF–Gaussian model to potential eruption of Mt. Baekdu

In order to examine the applicability of PUFF-Gaussian model for larger eruption, a hypothetical eruption of Mt. Baekdu located at the border of China and North Korea was tested by using both PUFF-UAF and the present method. It has been well known that Mt. Baekdu has tens of eruptions over more than as thousand years and recorded a historic eruption at VEI 7 in 946 AD^1^.

**Figure 16:**
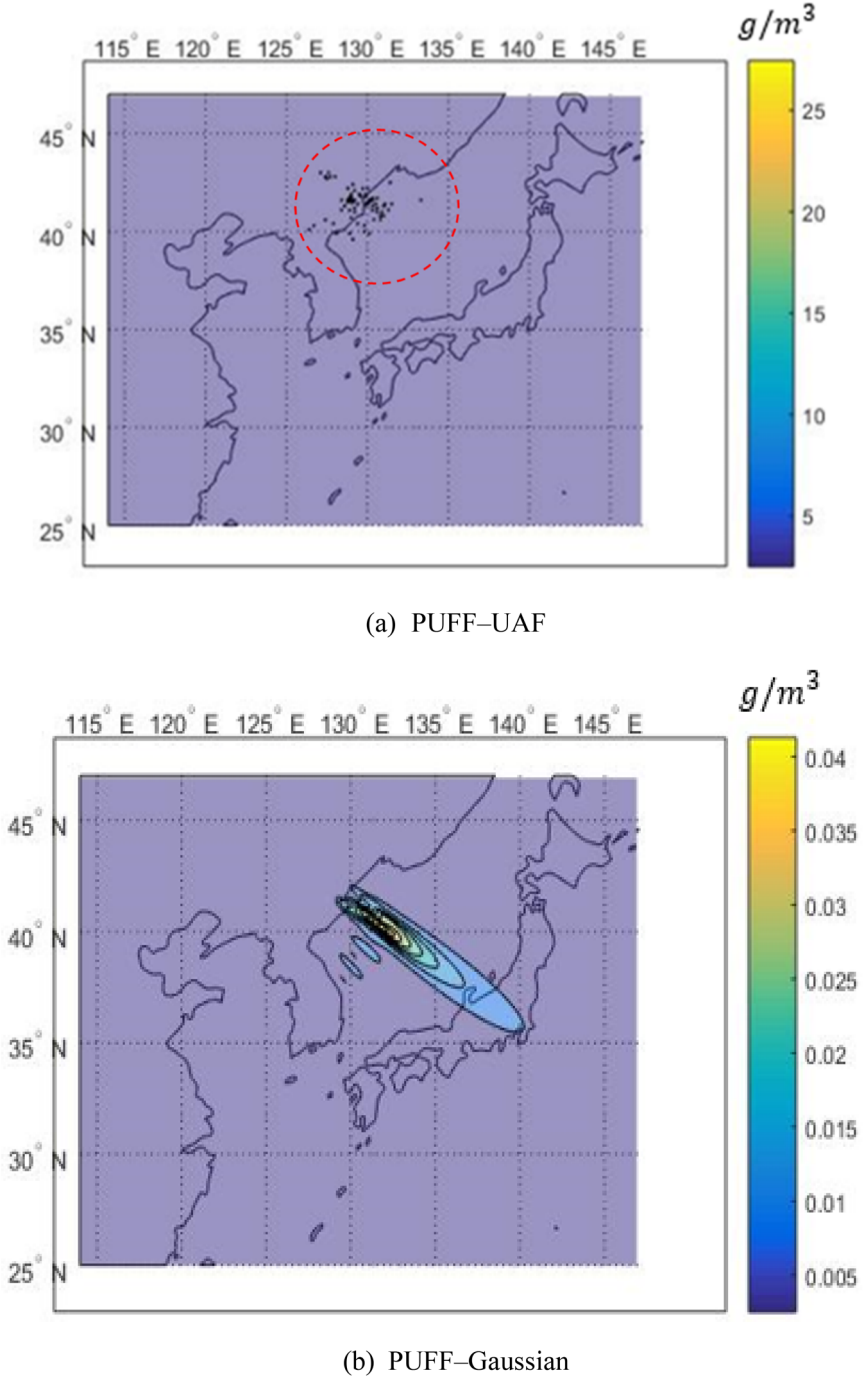
Comparison of ash concentrations at ground level between (**a**) PUFF–UAF and (**b**) PUFF–Gaussian models at 36 h after a hypothetical eruption of Mt. Baekdu on Sep. 7th, 2010 of VEI = 7.

The hypothetical eruption was assumed to occur on September 7, 2010 at VEI 7 with 100 ${\mathrm{km}}^{3}$ as the volume of particles. Figure 16 shows volcanic ash concentrations near the ground at 36 h after the eruption.

As shown in Fig. 16a, the dispersion result of the PUFF–UAF model depicts the problems of the Lagrangian method described in the previous section. Specifically, the concentration distribution is discontinuous and additionally indicates that the maximum concentration value is 25 $\mathrm{g}/{\mathrm{m}}^{3}$ or more. Figure 16b shows the results of applying the PUFF–Gaussian model which results in smooth and continuous distribution of the ash concentration with the maximum value of 0.04 $\mathrm{g}/{\mathrm{m}}^{3}$.

The difference in the maximum concentration is more than 600 times between two methods. It is because PUFF–UAF model dispersed a limited number of particles which was concentrated at a specific location, whereas PUFF–Gaussian model dispersed and diffuses the volcanic ash to a large area around the concentration point by the influence of mass diffusion.

## Discussion and conclusions

In this study, the Lagrangian-based PUFF–UAF model for predicting concentrations within diffusions of particles, such as volcanic ash, was compared with the newly developed PUFF–Gaussian model; this latter model was developed to apply the Gaussian dispersion model to the results of the PUFF–UAF model and improve the latter’s accuracy.

The results can be summarized as follows:

1. The problem of a discontinuously distributed volcanic ash diffusion, which is demonstrated by the Lagrangian model's particle number setting, and the problem of high concentration calculations at a specific location according to the particle number were addressed through a newly developed PUFF–Gaussian model. The proposed model applied a Gaussian dispersion model to the results of the PUFF–UAF model, thereby complementing the results of the Lagrangian-based model.
2. For the verification of the PUFF–Gaussian model, the results of the actual eruption of Shinmoedake and Ontake Volcanoes were compared.
3. During the development of the PUFF–Gaussian model, the concentration estimated by the Lagrangian method was assumed to be diffused by the mass diffusion process applying the Gaussian dispersion theory, and the results were valid.
4. Moreover, we identified areas that were not previously considered for heavy volcanic ash deposits surrounding the crater. We expect to increase the accuracy of the proposed model through further research and modification.
